# Clinical Observation of Factors in the Efficacy of Blood Component Transfusion in Patients following Hematopoietic Stem Cell Transplantation

**DOI:** 10.1371/journal.pone.0036912

**Published:** 2012-05-18

**Authors:** Xi Zhang, Yanni Xiao, Qian Ran, Yao Liu, Qianbi Duan, Huiling Duan, Xingde Ye, Zhongjun Li

**Affiliations:** 1 Department of Blood Transfusion, Xinqiao Affiliated Hospital of the Third Military Medical University, Chongqing, China; 2 Department of Hematology, Xinqiao Affiliated Hospital of the Third Military Medical University, Chongqing, China; French Blood Institute, France

## Abstract

**Background:**

Factors affecting the efficacy of platelet and red blood cell (RBC) transfusion in patients undergoing hematopoietic stem cell transplantation (HSCT) have not been studied extensively. We aimed to evaluate platelet and RBC transfusion efficacy by measuring the platelet corrected count increment and the hemoglobin increment, respectively, 24 h after transfusion in 105 patients who received HSCT.

**Methodology/Principal Findings:**

Using retrospective analysis, we studied whether factors, including gender, time of transplantation, the compatibility of ABO group between HSC donors and recipients, and autologous or allogenic transplantation, influence the efficacy of blood component transfusion. We found that the infection rate of HSCT patients positively correlated with the transfusion amount, and the length of stay in the laminar flow room was associated with transfusion. We found that platelet transfusion performed during HSCT showed significantly better efficacy than that performed before HSCT. The effect of platelet transfusion in auto-transplantation was significantly better than that in allo-transplantation. The efficacy of RBC transfusion during HSCT was significantly lower than that performed before HSCT. The efficacy of RBC transfusion in auto-transplantation was significantly higher than that in allo-transplantation. Allo-transplantation patients who received HSCs from compatible ABO groups showed significantly higher efficacy during both platelet and RBC transfusion.

**Conclusions:**

We conclude that the efficacy of platelet and RBC transfusions does not correlate with the gender of patients, while it significantly correlates with the time of transplantation, type of transplantation, and ABO compatibility between HSC donors and recipients. During HSCT, the infection rate of patients positively correlates with the transfusion amount of RBCs and platelets. The total volume of RBC units transfused positively correlates with the length of the patients’ stay in the laminar flow room.

## Introduction

Hematopoietic stem cell transplantation (HSCT) refers to a type of transplantation in which the patient is pretreated with total body irradiation (TBI), chemotherapy, and immunosuppressive pre-therapy followed by the transfusion of HSCs from normal donors or from the patient himself/herself through blood vessels [1]. Transfused HSCs possess the ability to proliferate, differentiate into functional mature blood cells, and retain their self-renewal capability. Hence, it can help patients rebuild normal hematopoietic and immune function [2], [3]. Based on whether HSCs come from healthy donors or the patient himself/herself, HSCT can be categorized into allogeneic (allo-) and autologous (auto-) HSCT [4]. After allo-HSCT, due to various reasons (such as chemotherapy and cell quiescent state), the platelet count and hemoglobin (Hb) value decreases, and transfusion of platelets and red blood cells (RBCs) in large amounts is needed [5]. Platelet transfusion is an effective method for treating different bleedings that are caused by reduced platelet [6], [7]. RBC transfusion can increase Hb levels and correct anemia [8]–[10]. Although the need for blood component transfusion in HSCT patients has increased rapidly, the guideline or strategy for clinical blood component transfusion practices in patients with HSCT remains unclear. It is unclear whether more amounts of transfusion would be better for HSCT patients. It is unclear whether transfusion would increase the infection rate in HSCT patients. It is unclear whether the length of HSCT patients’ stay in the lamina flow room would be affected by transfusion, and it is also unclear whether the compatibility of HSCs would have some effect on the transfused RBCs and platelets. Factors that influence the efficacy of blood component transfusion during HSCT and the role of blood transfusion in clinical outcomes in patients undergoing HSCT remains poorly understood. The aim of this study is to gain more insight and knowledge about blood component transfusion practices in populations undergoing HSCT.

## Results

### Efficacy of Platelet Transfusion in Patients Following HSCT

**Figure 1:**
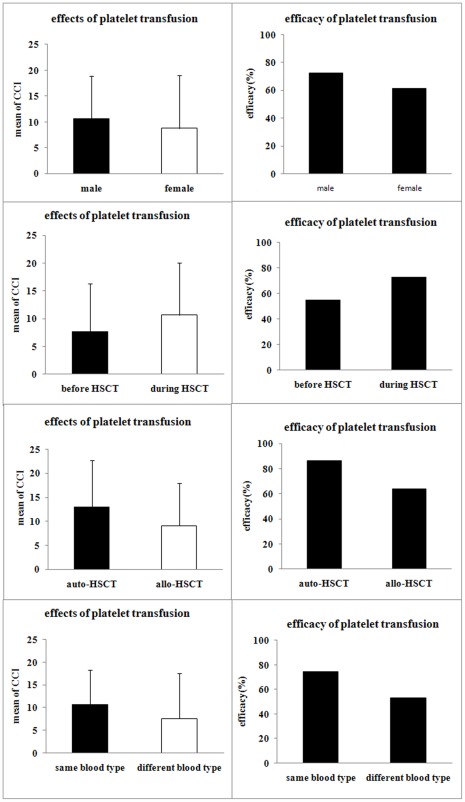
Influential factors of the effect of platelet transfusion in patients with HSCT. We investigated factors influencing the effect of platelet transfusion, including gender (A, B), the time of transplantation (C, D), auto-transplantation and allo-transplantation (E, F), and compatibility of blood types the between recipients and donors (G, H). The error bar represents standard deviation. The results are shown as mean ± S.D.

Among the 105 patients, the efficacy of platelet transfusion in male and female HSCT patients was 72.47% and 61.27%, respectively, and gender did not correlate with the efficacy of platelet transfusion (t = 1.844, p = 0.066, Figure 1 A and 1 B). Platelet transfusion during HSCT showed a significantly higher efficacy (72.69%) than transfusion before HSCT (54.84%; t = 2.612, p = 0.009, Figure 1 C and 1 D). The effect of platelet transfusion during auto-transplantation was significantly better than that during allo-transplantation (86.52% vs. 63.77%, t = 3.102, p = 0.002, Figure 1 E and 1 F). The effect of platelet transfusion in recipients who received compatible HSCs was better than that in recipients who received incompatible HSCs (74.29% vs. 52.94%, t = 2.034, p = 0.044, Figure 1 G and 1 H).

### Efficacy of RBC Transfusion in Patients Following HSCT

**Figure 2:**
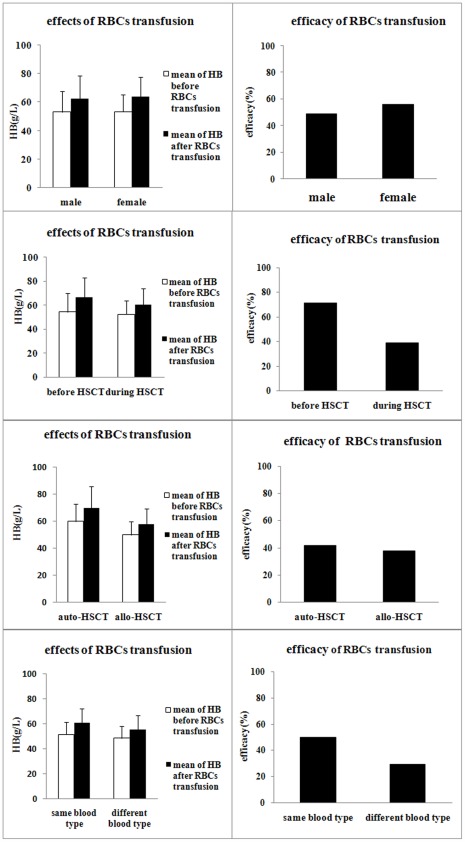
Influential factors of the effect of RBC transfusion in patients with HSCT. We investigated factors influencing the effect of RBC transfusion, including gender (A, B), the time of transplantation (C, D), auto-transplantation and allo-transplantation (E, F), and compatibility of blood types between the recipients and donors (G, H). The error bar represents standard deviation. The results are shown as mean ± S.D.

Among the 105 patients, the efficacy of RBC transfusion ranged 29.09–71.21%. The gender of the patients did not correlate with the efficacy of RBC transduction (49.18% vs. 56.25%, p = 0.684, Figure 2 A and 2 B). The effect of RBC transfusion during HSCT was significantly lower than that before HSCT (38.71% vs. 71.21%, p = 0.035, Figure 2 C and 2 D). The effect of RBC transfusion during auto-transplantation was significantly better than that during allo-transplantation (41.94% vs. 37.63%, p<0.001, Figure 2 E and 2 F). The effect of RBC transfusion when the HSC donor and recipient had the same blood type was better than when they had different blood types (50.00% vs. 29.09%, p = 0.044, Figure 2 G and 2 H).

### Relationship Between Infection Rate and Transfusion

**Figure 3:**
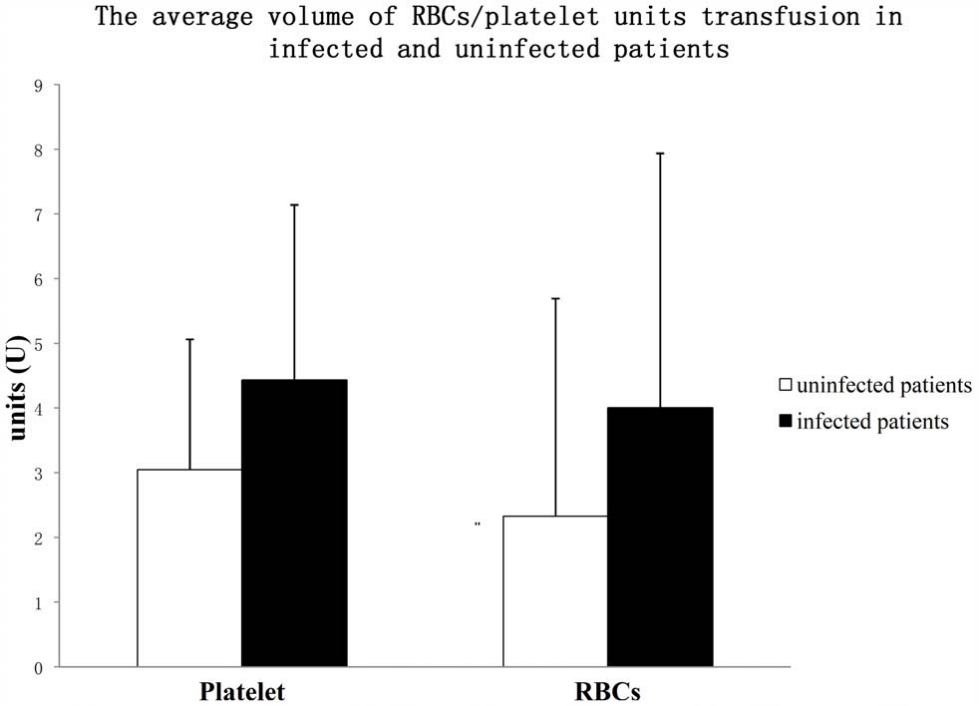
The average volume of platelet and RBC units transfused in patients with or without infections during HSCT. The total volume of platelet and RBC units transfused both positively correlated with the infection rate. The error bar represents standard deviation. The results are shown as mean ± S.D.

The average volume of platelet units transfused in patients who had infections during HSCT was greater than that in patients who had no infection during HSCT (4.431 U vs. 3.045 U), and we found that the total volume of platelet units transfused positively correlated with the infection rate (R^2^ = 0.288, p<0.05). The average volume of RBC units transfused in patients who had infections during HSCT was also greater than that in patients who had no infection (4.000 U vs. 2.328 U). The total volume of RBC units transfused also positively correlated with the infection rate (R^2^ = 0.247, p<0.05, Figure 3).

### Relationship Between Factors and the Length of Patients’ Stay in the Laminar Flow Room

Regression analysis showed that during HSCT, the total volume of RBC units transfused positively correlated with the length of the patients’ stay in the laminar flow room (R^2^ = 0.244, F = 32.641, p<0.001), whereas the volume of RBC units transfused and the number and total amount of platelets transfused did not correlate with the length of patients’ stay in the laminar flow room.

In addition, we also investigated the relation among the efficacy of RBC transfusion, age, major diseases, and graft-versus-host disease (GVHD). Using rank analysis, we found that these 3 factors did not correlate with the efficacy of RBC transfusion (R^2^ = 0.153, p = 0.090; R^2^ = 0.064, p = 0.481; and R^2^ = 0.137, p = 0.130, respectively). Major diseases correlated with platelet transfusion (R^2^ = 0.169, p<0.05), but age and GVHD did not correlate with platelet transfusion (R^2^ = 0.090, p = 0.179 and R^2^ = 0.067, p = 0.312, respectively).

## Discussion

In this study, we found that the efficacy of platelet transfusion during HSCT and in patients with auto-transplantation and matched ABO blood type between HSC donors and recipients was significantly higher than that of transfusion before HSCT and in patients with allo-transplantation and unmatched ABO blood type, respectively. The RBC transfusion was more effective when it was performed before HSCT and in patients with auto-transplantation and matched ABO blood type between HSC donors and recipients. Also, the infection rate in HSCT patients positively correlated with the average transfusion amount, and the total volume of RBC units transfused positively correlated with the length of the patients’ stay in the laminar flow room, whereas the number of RBC units transfused and the number and total amount of platelet transfusion were not correlated. These data provide guidance for future blood component transfusion practices in HSCT patients.

### Evaluating the Effect of Platelet Transfusion in HSCT Patients

Platelet transfusion is an effective method for treating bleeding caused by platelet decrement or platelet function deficiency and can reduce death rate caused by bleeding due to platelet decrease after radiation therapy or chemotherapy [11], [12]. The currently used apheresis platelet from a single donor not only decreases the danger of transmitting blood-borne diseases and the chance of homoimmune reaction, but also has homogeneous immunological characteristics. Clinically, the demand for apheresis platelet from a single donor is increasing every year. However, until now there have not been well-accepted specific standards for evaluating its clinical application in patients with HSCT. In some patients, expected results were not achieved after platelet transfusion, and after repeated transfusion, the efficacy of platelet transfusion significantly reduced. Previous reports have shown that ineffective platelet transfusion was 30–70% [13], [14].

Factors that influence platelet transfusion include non-immunologic factors such as platelet quality, infection, disseminated intravascular coagulation, bone marrow transplantation, and splenomegalia and immunologic factors such as repeated platelet transfusion that causes the production of human leukocyte antigen (HLA)-I and human platelet antigen (HPA) antibodies [15], [16]. Afterward, more platelet transfusion leads to an antigen-antibody reaction and damages the transfused platelets, which decreases the platelet count and might result in symptoms such as chills and fever [17], [18]. In this study, during HSCT, the patients repeatedly received platelet transfusion, and the number of platelet transfusions received was many times more than that before HSCT. Theoretically, the efficiency of platelet transfusion during HSCT should not be as good as that before HSCT, while the results showed an opposite outcome (see Table 1). This might be related to the pretreatments during the preparation for HSCT. To minimize graft rejection after HSCT, immune functions were suppressed in the recipients by pretreatments involving TBI and administration of cytotoxic drugs and immunosuppressors. Immunosuppression weakens phagocytic function of the spleen that eliminates transfused platelets and causes ineffective platelet transfusion, especially in patients with splenomegalia. In this study, all patients who received HSCT had pretreatments with immunosuppressives, and this might explain the high efficacy of platelet transfusion observed during HSCT and the cause of infection after transfusion.

**Table 1:** Efficacy of platelet transfusion in105 patients following HSCT.

		Number of transfusion	Number of effective transfusion	Efficacy (%)	
Gender	Male	178	129	72.47	*t* = 1.844, *p* = 0.066
	Female	142	87	61.27	
Time of transfusion	Before transplantation	93	51	54.84**	*t* = 2.612, *p* = 0.009
	During transplantation	227	165	72.69	
Type of transplantation	Auto-transplantation	89	77	86.52**	*t* = 3.102, *p* = 0.002
	Allo-transplantation	138	88	63.77	
Blood types of donor and recipient	Same	70	52	74.29*	*t* = 2.034, *p* = 0.044
	Different	68	36	52.94	

Note: The “effective transfusion” refers to those with CCI≥4.500 24 h after transfusion; number of units transfused Plt1U/transfusion;

* p<0.05;

** p<0.01;

*** p<0.0001.

In this study, we also observed that ABO compatibility between HSC donors and recipients correlated with efficacy of platelet transfusion. The blood-type incompatibility in transplantation could be a major ABO incompatibility, minor ABO incompatibility, or both. Transfusion of a small amount of RBCs mixed with platelets (there are ABH antigens on the surface of platelets) in HSCT patients receiving incompatible ABO blood type could increase the ineffective rate [19]. Marktel *et al.* [20] found that 76% successful platelet transfusions were obtained in HLA-matched and ABO-compatible patients followed by 67% in HLA-1-mismatched and ABO-compatible patients or HLA-matched and ABO-incompatible patients and by 46% in HLA-1-mismatched and ABO-incompatible patients. Our observation was consistent with this report. Therefore, compatibility of ABO blood types between HSC donors and recipients is very important for platelet transfusion in patients receiving HSCT, and the potential acute hemolytic transfusion reaction should be closely monitored.

### Evaluating the Effect of RBC Transfusion in HSCT Patients

RBC transfusion increases Hb concentration and alleviates the oxygen-deficient condition of the organism. Our data showed that the overall efficacy of RBC transfusion in HSCT patients was 52.29%, which was lower than the efficacy of RBC transfusion reported in literature. There might be 2 reasons for this low efficacy of RBC transfusion in HSCT patients. First, most of the transfusion occurred after HSCT with an average efficacy of 38.71%, which is much lower than that before HSCT (71.21%; Table 2). The efficacy of RBC transfusion before HSCT was still very close to the 80% reported in literature. Secondly, within some time after HSCT, the original hematopoietic system of the patients was destroyed, while the new grafted hematopoietic system was not functional; therefore, the effect of RBC transfusion during this period was worse than that before HSCT.

**Table 2:** Efficacy of red blood cell transfusion in 105 patients following HSCT.

		Number of transfusion	Number of effective transfusion	Efficacy (%)	
Gender	Male	122	60	49.18	*F = 0. 167, P = 0.684*
	Female	96	54	56.25	
Time of transfusion	Before transplantation	94	66	71.21*	*F = 4.512, P = 0.035*
	During transplantation	124	48	38.71	
Type of transplantation	Auto-transplantation	31	13	41.94***	*F = 20.307,* *P<0.001*
	Allo-transplantation	93	35	37.63	
Blood types of donor and recipient	Same	38	19	50.00*	*F = 4.155, P = 0.044*
	Different	55	16	29.09	

Note: The “effective transfusion” refers to those with Hb elevated by 10 g 24 h after transfusion of 2U red cell suspension;

* p<0.05;

** p<0.01;

*** p<0.001.

In this study, we found that ABO blood-type compatibility between the HSC donor and recipient significantly correlated with RBC transfusion efficacy. When allo-HSCT patients receive RBC transfusion from a major ABO incompatible blood source, the rebuild of the erythroid system was found to be slow [21], [22]. However, the incidence rate of GVHD was not significantly affected [23]. In about 15–30% of all the allo-HSCTs, the HLA types but not the ABO blood types of the donor and recipient were compatible [24], [25]. Although it had been recommended early that HSCT should not be performed using ABO incompatible donors, it was shown later that the ABO blood-type system is not an important transplantation antigen system. The ABO-blood type compatibility did not significantly correlate with the successful grafting of HSC, graft rejection, or survival of the patients, nor did it substantially affect the incidence and severity of GVHD. Therefore, the ABO blood type is not a contraindication of HSCT. However, ABO blood-type incompatibility of the donor and HSCT recipient may lead to severe platelet transfusion refractoriness (PTR) [26]. Consequently, during HSCT, the HSC suspension or the recipient should be processed properly, and attention should be paid to blood transfusion after transplantation. Clinical practice of over 20 years has shown that with an appropriate approach, allo-HSCT can be successfully performed despite ABO blood-type incompatibility. In allo-bone marrow transplantation with ABO blood-type incompatibility, procedures that decrease antibody titer of the recipient to the donor blood-type antigen should be carried out to avoid acute hemolytic transfusion reaction; however, in this type of transplantation, large amounts of RBC transfusion is needed, and the restoration of the erythroid system is slow [27]. Hence, blood component transfusion will be more important in patients following the ABO-incompatible donor’s HSCT. Compared to bone marrow transplantation, peripheral HSCT promotes the recovery of the erythroid system. It has been considered that even in the presence of anti-donor antibody, large amounts of erythroid progenitors in the peripheral blood grafts may promote restoration of the erythroid system, and ABO blood-type incompatibility does not affect the successful grafting of allo-HSCT and prognosis [28].

Based on previous results, we have noticed that transfusion of RBCs and platelets was greater in infected patients than in uninfected patients undergoing HSCT. Due to immune dysfunction after HSCT, cytomegalovirus-specific cytotoxic T cells and T helper cells do not respond effectively, and they cannot clear the infection and generate immune protection, resulting in latent in vivo viral recrudescence or cytomegalovirus infection through transfused cells. In clinical practice, if we are free to raise the transfusion threshold and use blood products excessively of patients, especially during HSCT, they will be exposed to a high risk of infection [29], [30] The length of patients’ stay in a laminar flow room increases with the number of total units of the RBC received. This may be explained by the fact that the increase in transfusion amount also raises the chances of infection or other diseases.

Our results also have showed that factors such as age, major diseases, and GVHD do not correlate with the efficacy of RBC transfusion. However, the efficacy of platelet transfusion correlated with the disease diagnosed when the patient was admitted for transfusion. This correlation may be explained by the fact that the damage caused by major diseases to hematopoiesis and immune function is different. After HSCT and chemotherapy, the recovery of the megakaryocyte system in patients is often slower than that of the RBC system, and the recovery of immune function is also slower than that of hematopoietic function.

### Transfusable RBC and Platelet Blood Types When the Blood Types of the Donor and the Recipient were Different During Peripheral HSCT

As the immune function is suppressed and the hematopoietic function is rebuilt in patients after HSCT, the effects of platelet and RBC transfusion are substantially different from those before HSCT. It might not be accurate to evaluate the efficacy of transfusion only based on the value calculated from the formulas for Charlson Comorbidity Index (CCI), percentage of platelet recovery, and Hb increment. Rather, it might be of more clinical significance to evaluate the transfusion effect based on a combination of values and other clinical outcomes of the patients after transfusion, and this question will need further study.

### Optimal use of Blood Components Requires More Attention

A declining donation rate and an increase in the consumption of blood components require improved approaches on the blood supply chain. Complex therapeutic procedures like HSCT may require intensive blood component support, which leads to a steady increase in the consumption of standard blood components, while blood component transfusions are critical for recovery in patients receiving HSCT. Therefore, alternative strategies that improve transfusion efficiency will decrease the blood product consumption per individual procedure.

As RBC antigen is a natural antibody, the transfusion efficacy might decrease when an incompatible ABO blood type is used for transfusion [31]. We propose to use transfusable, washed blood type O (Table 2) to reduce the demand for RBC transfusion and the risk of transfusion-transmitted disease and ensure the support of oxygen in tissues during the HSCT. It might also decrease the total amount of transfused RBCs during transplantation as well as the length of stay in the laminar flow room.

During HSCT, immune function in patients is suppressed and immune-related platelet transfusion is more efficient. So, in combination with clinical manifestations, the threshold for platelet transfusion can be reduced from 20×10^9^/L to 10×10^9^/L or even less, which could save blood for transfusion, relieve the pressure on blood supply, decrease the financial burden of patients, and ensure clinical efficacy at the same time.

In summary, our data have shown a relationship between the clinical outcome of the blood component transfusion and factors that affect the efficacy of blood component transfusion. A sound clinical transfusion plan is needed not only to reduce the blood product consumption, but also to reduce the risk of infections.

## Materials and Methods

### Subjects

A total of 105 patients were randomly selected from among patients who were admitted to the Hematology Department of the Xinqiao Hospital affiliated to the Third Military Medical University (Chong Qing, China) between January 1, 2006, and August 31, 2010. These patients suffered from platelet decrease and anemia before HSCT (day −60–day 0) and during HSCT (day 0–day 52). Patient and HSCT characteristics are summarized in Table 3. Written informed consent was obtained from each patient, and this study was approved by the ethics committee of the Xinqiao Affiliated Hospital of the Third Military Medical University of Chinese of the People’s Liberation Army (PLA).

**Table 3:** Patients and HSCT characteristics.

	Number of patients	%
**Gender**		
Male	62	59.05
Female	43	40.95
**Donor type**		
Auto-transplantation	41	39.051
Allo-transplantation	64	60.95
**ABO groups of HSC donors and recipients**		
Match	38	59.38
Mismatch	26	40.62
**Main complications**		
Bleeding gums	8	7.62
Canker sore and nasal cavity bleeding	40	38.10
Petechial hemorrhage spots on the skin	22	20.95
Gastrointestinal bleedinginfected patients	736	6.6734.29
**Primary diagnosis**		
Acute lymphoblastic leukemia	13	12.38
Acute non-lymphocytic leukemia	24	22.86
Chronic myelocytic leukemia	18	17.14
Aplastic anemia	3	2.86
Thrombopenic purpura	3	2.86
Non-Hodgkin’s lymphoma	20	19.05
Hodgkin’s lymphoma	4	3.81
Lymphoma leukemia	6	5.71
Multiple myeloma	3	2.86
Neuroendocrine tumor	1	0.95
Hybrid acute leukemia	2	1.90
Myeloproliferative disease	3	2.86
Systemic lupus erythematosus	2	1.90
Hand-schüller-christian disease	1	0.95
Anaplastic large-cell lymphoma	1	0.95
Primary macroglobulinemia	1	0.95
**Median Age at HCT (range), years**	33 (3∼68)	
**Total number**	105	

### Equipment and Blood Components

BC-5500 and BC-5300 hematology analyzers (Mindray Inc.), XT 1800i and SE-9000 hematology analyzers (Sysmex), CS-3000 plus blood cell separator (Baxter), and Fresenius COM.TEC blood cell separator (Fresenius) were used in this study. Apheresis platelets and RBC suspensions were provided by the Chongqing Blood Center.

### Evaluation Standards for the Effect of Blood Component Transfusion

#### Platelets

Platelet corrected count increment (CCI)  =  platelet increment (PI, 10^9^/L) × body surface area of the recipient (BSA; m^2^)/total number of transfused platelets (10^11^/L). The BSA of the recipient (m^2^) = 0.0061× height (cm) +0.0128× weight (kg) − 0.01529. For 1 U, the number of single donor platelets was 2.5×10^11^/bag, and it is same for all the formulas given below [2]. The platelet transfusion was considered ineffective if CCI <7.5 1 h after the transfusion and <4.5 20–24 h after transfusion. Due to limited condition, very few patients in our study were tested for CCI 1 h after platelet transfusion; hence, the evaluations were all based on CCI 24 h after transfusion [32], [33]. We investigated the impact of several factors, including gender, time of transfusion following HSCT, auto- or allo-transplantation, and ABO blood-type compatibility between HSC donors and recipients on the efficacy of platelet transfusion. Patients underwent transfusion if their platelet threshold was 10×10^9^/L [34], [35] or if they had severe bleeding.

#### RBCs

To evaluate the effect of RBC transfusion, we considered 3 standards, including RBC count, hematocrit (Hct), and Hb. As the main purpose of RBC transfusion is to elevate Hb to carry more oxygen, the most important index for calculating the clinical effect of RBC transfusion is Hb increment in circulating blood. The other 2 factors are often used as references. In general, 24 h after transfusion of 2 U (400 mL) of RBC suspension, the Hb level increases by 10 g/L. The RBC transfusion was considered ineffective if Hb increment was lower than 10 g/L. According to the ‘Technical Guide for Clinical Transfusion’ in China, we performed transfusion if a patient had Hb <60 g/L or had symptoms such as severe bleeding or anemia.

For incompatible ABO blood type between HSC donors and recipients, the blood type of transfusable washed RBCs and platelets was according to transfusion compatibility chart (Table 4).

**Table 4:** Transfusion compatibility chart for incompatible ABO type of donor and recipient (Weissinger *et al,* 2011).

Donor blood type	Recipient blood type	Blood type of transfusable washed RBCs	Blood type of transfusable apheresis platelets
A	B	O	AB
A	O	O	AB/A
A	AB	O/A	AB
B	O	O	AB/B
B	AB	O/B	AB
O	A	O	A/AB
O	B	O	B/AB
O	AB	O	AB
AB	A	O/A	AB
AB	B	O/B	AB

### Statistical Analysis

Statistical Package for the Social Sciences (SPSS) 18.0 software was used for statistical analysis. Student’s *t*-test was performed to analyze the significance of the efficacy of platelet transfusion. Repeated measures analysis of variance was performed to analyze the significance of the efficacy of RBC transfusion. Spearman’s rank correlation analysis was used to analyze the relationship between the infection rate and the total amount of platelet and RBC transfusion during HSCT. Multiple regression (stepwise method) was adopted for statistical analysis of the relation among the number and total amount of RBC and platelet transfusion, Hb concentration at the first transfusion, and the patient’s length of stay in the laminar flow room. Results with p<0.05 were considered statistically significant.
